# Assessment of emotions and behaviour by the Developmental Behaviour Checklist in young people with neurodevelopmental CNVs

**DOI:** 10.1017/S0033291720002330

**Published:** 2022-02

**Authors:** Adam C. Cunningham, Jeremy Hall, Stewart Einfeld, Michael J. Owen, IMAGINE-ID consortium, Marianne B. M. van den Bree

**Affiliations:** 1 Division of Psychological Medicine and Clinical Neurosciences, MRC Centre for Neuropsychiatric Genetics and Genomics, Cardiff University School of Medicine, Cardiff, UK; 2 Faculty of Health Sciences, University of Sydney, Sydney, Australia

**Keywords:** Behaviour, cnv, genetics, intellectual disability, psychopathology

## Abstract

**Background:**

A number of genomic conditions caused by copy number variants (CNVs) are associated with a high risk of neurodevelopmental and psychiatric disorders (ND-CNVs). Although these patients also tend to have cognitive impairments, few studies have investigated the range of emotion and behaviour problems in young people with ND-CNVs using measures that are suitable for those with learning difficulties.

**Methods:**

A total of 322 young people with 13 ND-CNVs across eight loci (mean age: 9.79 years, range: 6.02–17.91, 66.5% male) took part in the study. Primary carers completed the Developmental Behaviour Checklist (DBC).

**Results:**

Of the total, 69% of individuals with an ND-CNV screened positive for clinically significant difficulties. Young people from families with higher incomes (OR = 0.71, CI = 0.55–0.91, *p* = .008) were less likely to screen positive. The rate of difficulties differed depending on ND-CNV genotype (χ^2^ = 39.99, *p* < 0.001), with the lowest rate in young people with 22q11.2 deletion (45.7%) and the highest in those with 1q21.1 deletion (93.8%). Specific patterns of strengths and weaknesses were found for different ND-CNV genotypes. However, ND-CNV genotype explained no more than 9–16% of the variance, depending on DBC subdomain.

**Conclusions:**

Emotion and behaviour problems are common in young people with ND-CNVs. The ND-CNV specific patterns we find can provide a basis for more tailored support. More research is needed to better understand the variation in emotion and behaviour problems not accounted for by genotype.

## Introduction

Many copy number variants (CNVs), caused by reciprocal deletions and duplications of chromosomal regions such as 1q21.1, 16p11.2 or 22q11.2, are associated with the development of neurodevelopmental disorders (hereafter referred to as ND-CNVs) (Chawner et al., 2019; Torres, Barbosa, & Maciel, 2015). These ND-CNVs are associated with a broad psychiatric and physical phenotype, often with variable expressivity and incomplete penetrance. This means that while some individuals with ND-CNVs are affected by many complex physical and mental health symptoms, others may display few or none (Crawford et al., 2018). As the technology used to detect these changes continues to improve, the rate of diagnosis of ND-CNVs is increasing. Therefore, there is a need to advance our understanding of the clinical outcomes associated with these genetic conditions. This will allow clinicians to provide optimal counselling and intervention to patients and families.

There is abundant evidence that individuals with ND-CNVs have higher rates of neurodevelopmental and psychiatric disorders than the general population (Bernier et al., 2016; Chawner et al., 2019; Glassford, Rosenfeld, Freedman, Zwick, & Mulle, 2016; Hanson et al., 2014; Schneider et al., 2014; Vermeulen et al., 2017). In addition to this, in individuals with ND-CNVs some disorders may show patterns of symptoms that differ from the classical presentation. For example, in individuals with 22q11.2DS, attention deficit-hyperactivity disorder is often of the inattentive subtype, rather than the hyperactive or combined subtypes that are more common in children without the deletion (Niarchou, Martin, Thapar, Owen, & van den Bree, 2015). In addition to these differences, there are often complex patterns of co-occurring symptoms, both physical and psychological, with evidence suggesting that there is an increased risk of psychiatric disorders in children with ND-CNVs who also display motor coordination problems (Cunningham et al., 2018), a history of seizures (Eaton et al., 2019), or sleep disturbances (Moulding et al., 2019). ID is common in individuals with ND-CNVs but can range from mild to severe (Chawner et al., 2019; Hanson et al., 2014; Schneider et al., 2014; Vissers, Gilissen, & Veltman, 2016), with evidence that the IQ of family members explains part of this vulnerability (Hanson et al., 2014).

Assessing mental health status in individuals with ID can be difficult (Matson & Shoemaker, 2011). Research studies often use standardized assessments of psychiatric disorder that follow the DSM or ICD classification systems. While these assessments allow for common discourse between researchers, they may not adequately assess disorders that present differently or are easily confused with symptoms of ID (Costello & Bouras, 2006).

This difficulty in assessing mental health status in individuals with ID has implications for studies investigating psychopathology associated with ND-CNVs, as participants are likely to have some degree of cognitive impairment. Despite this, few studies have used assessments of psychopathology that are tailored for individuals with ID (Einfeld, Tonge, & Florio, 1997; Vermeulen et al., 2017; Wagner, Niemczyk, Equit, Curfs, & von Gontard, 2017). Thus, behaviours that are more common in individuals with ID will not have been captured. The Developmental Behaviour Checklist (DBC) (Einfeld & Tonge, 1995) is designed to measure emotional and behavioural problems in young people with ID. Using the DBC in studies of individuals with ND-CNVs can, therefore, provide important insights into ID-specific behaviours that may have been missed in previous research.

In addition to this, few studies have conducted cross-CNV comparisons of emotion and behaviour problems in young people with ND-CNVs (Di Nuovo & Buono, 2011; Vermeulen et al., 2017). It is, therefore, unclear if there are differences in the severity of specific behaviours or emotional difficulties between individual ND-CNV genotypes. Knowing if these differences exist is important as it will influence what counselling or support should be provided.

To address the gaps in the literature surrounding emotion and behaviour disturbance profiles in young people with ND-CNVs, we assessed a sample of 322 young people with a range of 13 ND-CNVs, across eight loci, using the DBC (Einfeld & Tonge, 1995). We investigated the following questions: (1) What proportion of young people with an ND-CNV screen positive for clinically significant emotion and behaviour disturbance as measured using the DBC? (2) Are there differences in these disturbances between males and females with ND-CNVs? (3) Do health and pregnancy-related variables influence rates of clinically significant difficulties? (4) Which emotion and behaviour problems are particularly elevated in young people with ND-CNVs? (5) Are there differences between ND-CNV genotypes in risk for the presence of clinically significant difficulties?

## Methods

### Participants

A total of 322 participants with ND-CNVs (including one of 15q11.2 deletion, 15q13.3 deletion, 15q13.3 duplication, 16p11.2 deletion, 16p11.2 distal deletion, 16p11.2 duplication, 1q21.1 deletion, 1q21 duplication, 22q11.2 deletion, 22q11.2 duplication, 9q34.3 deletion (Kleefstra syndrome), NRXN1 (2p16.3 deletion) or TAR (1q21.1) duplication (66.5% male, mean age: 9.79 years, range: 6.02–17.91) assessed between September 2011 and November 2018 as part of the ECHO and IMAGINE-ID studies at Cardiff University. Participants were recruited on the basis of having an ND-CNV of interest, not the presence of ID or other neurodevelopmental disorder. Data from all instruments used were collected at the same time. Families were recruited through UK Medical Genetics clinics, word of mouth, and charities and support groups for chromosomal disorders including Unique, MaxAppeal! and 22qCrew. Informed and written consent was obtained prior to recruitment from the carers of the children, or the children themselves where appropriate. Recruitment was carried out in agreement with protocols approved by the appropriate university and National Health Service (NHS) ethics and research and development committees. Families were visited at home for phenotyping including cognitive and psychiatric assessments. ND-CNV genotypes were established from medical records as well as in-house genotyping at the Cardiff University MRC Centre for Neuropsychiatric Genetics and Genomics using microarray analysis. Information about the pregnancy and the children's medical history, including congenital heart defects, history of epilepsy as well as psychiatric and epilepsy medication use was collected through primary carer report. Psychiatric and epilepsy medication use is listed in online Supplementary Table 1.

### The developmental behaviour checklist

**Fig. 1.**
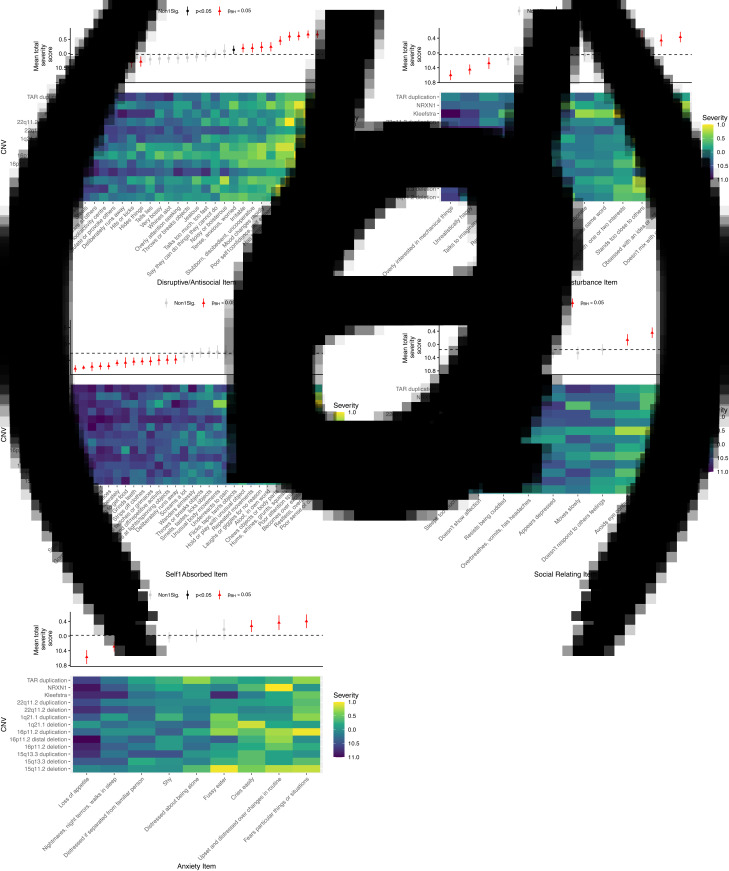
Heat plots of scores for the items constituting the five subscales of the DBC (*a*) disruptive/antisocial, (*b*) communication disturbance (*c*) self-absorbed (*d*) social relating and (*e*) anxiety. Items are ordered on the *x*-axis by mean total severity score for each question across the entire ND-CNV sample, from lowest on the left to highest on the right. The chart above each heat plot shows the mean total severity score and 95% confidence interval for each question. Point shape corresponds to the significance of results of a *t* test comparing the mean of the total severity score of the question to the overall mean for all items in the scale. Squares indicate a nonsignificant difference, circles indicate a nominally significant difference and triangles indicate questions that survived Benjamini-Hochberg correction.

We used the DBC, which was developed to assess emotion and behaviour problems in individuals with ID (Einfeld & Tonge, 1995). It has been used in studies of idiopathic ID (Einfeld et al., 2006) as well as studies of genetic syndromes associated with ID (Einfeld et al., 1997; Rice et al., 2016; Wagner et al., 2017). The DBC provides total behaviour problems score (TBPS) as well as scores on five subscales: disruptive/antisocial behaviour (27 items), self-absorbed behaviour (30 items), social relating (10 items), communication disturbance (13 items), and anxiety (9 items). Some items are present in more than one domain. Responses are coded as 0 = ‘not true’, 1 = ‘somewhat true’, 2 = ‘certainly true’. See Fig. 1 for all items that are included in each of these subscales. Two items, ‘masturbates or exposes self in public’ and ‘inappropriate sexual behaviour with another’ were not included in this study, because the research team deemed these less appropriate to ask the primary carer to complete by questionnaire. Thus, the DBC version we used included a total of 94 items. The DBC can also be used to screen for the presence of clinically significant emotion and behaviour difficulties, indicated by a TBPS greater than 45.

### Statistical analysis

Statistical analysis was carried out in R version 3.6.3 (Development Core Team, 2011). In total, 460/30 268 (1.5%) of total DBC responses were missing. These were assumed to be missing at random and imputed using *k-*nearest neighbour imputation with *k* = 5 using the ‘VIM’ package to create a complete dataset (Kowarik & Templ, 2016).

In order to address Aim 1, the percentage of individuals with an ND-CNV displaying clinically significant emotion and behavioural difficulties (TBPS >45) was calculated. As the presence of clinically significant emotion and behavioural difficulties is a dichotomous (yes/no) variable, we used logistic regression to investigate if age, gender, approximate family income and maternal education level were predictors of the presence of clinically significant emotion or behavioural difficulties.

For aim 2, we used a chi-squared test to learn if rates of screening positive for clinically significant difficulties differed by gender. We also used *t* tests to investigate if the continuous DBC total problems score and subscale scores differed by gender.

To address aim 3, we used logistic regression to investigate if health and pregnancy-related variables were associated with the presence or absence of clinically significant difficulties. We constructed models where the dichotomous variable ‘presence of clinically significant difficulties’ was predicted by the presence of congenital heart defects; a history of epileptic fits; being part of multiple births; whether the mother had fertility treatment during the pregnancy; or prolonged labour (longer than 36 h); the baby having spent time in a special care baby unit (SCBU); or in an incubator; or having been born prematurely (before 37 weeks for a single birth, or before 34 weeks for twins). These variables were included in addition to gender, age, maternal education and approximate family income.

In order to address aim 4, we plotted heatmaps of all the items comprising each DBC subdomain. This allowed us to investigate which emotion and behaviour problems impact relatively more or less severely on young people with ND-CNVs (Fig. 1*a*–*e*). In these heatmaps, each cell represents the deviation from the mean for each question and each ND-CNV genotype. For example, for the disruptive/antisocial subscale (Fig. 1*a*) the average score for each of the 27 items was calculated for each of the 13 ND-CNV genotypes giving a total of 351 values. The mean of these 351 values was calculated, and the deviation from the mean for each ND-CNV and item combination was plotted. Each cell, therefore, represents the deviation from the overall mean of the 351 values in the plot. To compare between different subscales (Fig. 1*a*–*e*) these deviations were linearly transformed to fall between 1 and −1 when plotted. In addition, to establish which items within each subscale are more or less common in young people with ND-CNV, we conducted *t* tests comparing the mean score across all ND-CNV groups for each item within a subscale with the mean score of all other items included in a subscale.

In order to address aim 5, we constructed a model with all covariates we found to be significant in the analysis of aims 1–3 (e.g. income) and added ‘ND-CNV genotype’ as an additional factor. We used a likelihood ratio test to establish if this additional factor contributed significantly to variation in DBC TBPS.

Furthermore, we used ANCOVAs to test if the continuous DBC-TBPS and scores on each of the five subscales differed by ND-CNV genotype, again including those covariates we had found to be predictors of the presence of clinically significant behavioural difficulties in aim 1 and aim 2. The pattern of severity across the TBPS and subscales was visualised (Fig. 1*a*–*e*) by plotting the marginal mean TBPS and subscale score for each ND-CNV genotype. These marginal means represent the predicted score after adjusting for those variables that were previously found to be predictors of the presence of clinically significant behavioural difficulties.

We also carried out ANCOVAs to investigate if the type of ND-CNV (deletion or duplication) or ND-CNV inheritance status (inherited from parent or *de novo*), were associated with TBPS, or scores on each of the five subscales. The association between inheritance status and ND-CNV genotype was tested with a chi-squared test of independence.

In order to test whether there are differences in the severity with which the different ND-CNVs impact on TBPS and the five subscales, we used Friedman's chi-square test. In addition, to test the concordance between scores on the scales, we used Kendall's *F*-test. Kendall's *F* test also provides a coefficient of concordance W. The coefficient of concordance allows us to assess the level of agreement in scores across ND-CNV genotypes. W can take values between 0 and 1, with 0 indicating no agreement in scores, and 1 indicating perfect agreement (Salkind, 2010). It can be interpreted as an effect size, with 0.1 ⩾ 0.3 corresponding to a small effect, 0.3 ⩾ 0.5 a moderate effect, and >0.5 a large effect.

The procedures outlined for aim 3 and aim 5 were repeated excluding those individuals who were taking psychiatric or epilepsy medication to establish if this would change the findings. For all analyses conducted, we checked to ensure they met assumption requirements.

To correct for multiple testing across the analyses, we applied a Benjamini–Hochberg correction of *p* values (Benjamini & Hochberg, 1995) using the *p.adjust* function in R, and report both original and adjusted *p* values throughout the Results section.

## Results

Table 1 describes the participants who took part in the study. Participants were aged from 6.01 to 17.91 years (mean age = 9.79) and 33.5% were female.

**Table 1.** Descriptive and summary statistics for the sample of children with neurodevelopmental copy number variants. Variables described are A) family background, B) demographics, C) Cognition, D) Developmental Behaviour Checklist scores and E) Health related variables.

	15q11.2 deletion (*N* = 36)	15q13.3 deletion (*N* = 20)	15q13.3 duplication (*N* = 10)	16p11.2 deletion (*N* = 52)	16p11.2 distal deletion (*N* = 13)	16p11.2 duplication (*N* = 23)	1q21.1 deletion (*N* = 16)	1q21.1 duplication (*N* = 21)	22q11.2 deletion (*N* = 70)	22q11.2 duplication (*N* = 23)	Kleefstra syndrome (*N* = 12)	NRXN1 (*N* = 16)	TAR duplication (*N* = 10)	Total (*N* = 322)
(*A*) *Family background*														
Maternal education														
N-Miss	0	0	0	0	0	1	0	0	1	1	0	0	0	3
Low (O-levels, GCSE's)	8 (22.2%)	7 (35.0%)	2 (20.0%)	18 (34.6%)	6 (46.2%)	4 (18.2%)	3 (18.8%)	5 (23.8%)	14 (20.3%)	3 (13.6%)	2 (16.7%)	7 (43.8%)	0 (0.0%)	79 (24.8%)
Middle (A-level's, Highers, vocational training)	14 (38.9%)	5 (25.0%)	5 (50.0%)	17 (32.7%)	4 (30.8%)	8 (36.4%)	6 (37.5%)	12 (57.1%)	31 (44.9%)	10 (45.5%)	5 (41.7%)	4 (25.0%)	6 (60.0%)	127 (39.8%)
High (University/ postgraduate degree)	9 (25.0%)	7 (35.0%)	3 (30.0%)	12 (23.1%)	3 (23.1%)	7 (31.8%)	4 (25.0%)	2 (9.5%)	20 (29.0%)	7 (31.8%)	5 (41.7%)	5 (31.2%)	2 (20.0%)	86 (27.0%)
No School Leaving Exams	5 (13.9%)	1 (5.0%)	0 (0.0%)	5 (9.6%)	0 (0.0%)	3 (13.6%)	3 (18.8%)	2 (9.5%)	4 (5.8%)	2 (9.1%)	0 (0.0%)	0 (0.0%)	2 (20.0%)	27 (8.5%)
Approximate family income														
N-Miss	4	1	1	10	1	3	2	1	7	2	1	2	0	35
< = £ 19 999	9 (28.1%)	4 (21.1%)	4 (44.4%)	14 (33.3%)	3 (25.0%)	10 (50.0%)	5 (35.7%)	12 (60.0%)	14 (22.2%)	8 (38.1%)	5 (45.5%)	3 (21.4%)	4 (40.0%)	95 (33.1%)
£ 20 000 - £ 39 999	11 (34.4%)	7 (36.8%)	2 (22.2%)	15 (35.7%)	6 (50.0%)	7 (35.0%)	8 (57.1%)	3 (15.0%)	14 (22.2%)	5 (23.8%)	1 (9.1%)	9 (64.3%)	6 (60.0%)	94 (32.8%)
£ 40 000 - £ 59 999	9 (28.1%)	3 (15.8%)	3 (33.3%)	10 (23.8%)	1 (8.3%)	2 (10.0%)	0 (0.0%)	3 (15.0%)	16 (25.4%)	6 (28.6%)	2 (18.2%)	2 (14.3%)	0 (0.0%)	57 (19.9%)
£ 60 000 +	3 (9.4%)	5 (26.3%)	0 (0.0%)	3 (7.1%)	2 (16.7%)	1 (5.0%)	1 (7.1%)	2 (10.0%)	19 (30.2%)	2 (9.5%)	3 (27.3%)	0 (0.0%)	0 (0.0%)	41 (14.3%)
(*B*) *Demographics of child participants*														
Child ethnicity														
N-Miss	0	0	1	0	0	0	0	0	0	1	0	0	0	2
European	33 (91.7%)	18 (90.0%)	9 (100.0%)	48 (92.3%)	13 (100.0%)	21 (91.3%)	15 (93.8%)	21 (100.0%)	64 (91.4%)	20 (90.9%)	11 (91.7%)	16 (100.0%)	10 (100.0%)	299 (93.4%)
Other	3 (8.3%)	2 (10.0%)	0 (0.0%)	4 (7.7%)	0 (0.0%)	2 (8.7%)	1 (6.2%)	0 (0.0%)	6 (8.6%)	2 (9.1%)	1 (8.3%)	0 (0.0%)	0 (0.0%)	21 (6.6%)
Age of child participants														
N-Miss	0	0	0	0	0	0	0	0	0	0	0	0	0	0
Mean (s.d.)	9.495 (3.018)	9.747 (3.123)	9.010 (2.840)	9.826 (3.256)	10.022 (3.298)	10.576 (3.480)	9.664 (2.781)	9.233 (3.377)	9.891 (2.863)	9.761 (3.240)	12.191 (3.911)	8.892 (2.773)	8.831 (2.590)	9.794 (3.119)
Range	6.022–16.380	6.169–15.419	6.413–15.186	6.068–17.477	6.135–16.750	6.197–17.280	6.079–15.829	6.016–17.060	6.019–17.905	6.297–16.480	6.255–17.577	6.160–15.667	6.594–14.092	6.016–17.905
Gender of child participants														
N-Miss	0	0	0	0	0	0	0	0	0	0	0	0	0	0
Female	8 (22.2%)	3 (15.0%)	2 (20.0%)	19 (36.5%)	4 (30.8%)	9 (39.1%)	6 (37.5%)	11 (52.4%)	27 (38.6%)	6 (26.1%)	7 (58.3%)	2 (12.5%)	4 (40.0%)	108 (33.5%)
Male	28 (77.8%)	17 (85.0%)	8 (80.0%)	33 (63.5%)	9 (69.2%)	14 (60.9%)	10 (62.5%)	10 (47.6%)	43 (61.4%)	17 (73.9%)	5 (41.7%)	14 (87.5%)	6 (60.0%)	214 (66.5%)
ND-CNV Inheritance status														
N-Miss	19 (52.8%)	12 (60.0%)	5 (50.0%)	36 (69.2%)	11 (84.6%)	17 (73.9%)	10 (62.5%)	14 (66.7%)	14 (20.0%)	12 (52.2%)	8 (66.7%)	9 (56.2%)	6 (60.0%)	173 (53.7%)
De Novo	1 (2.8%)	1 (5.0%)	1 (10.0%)	12 (23.1%)	2 (15.4%)	1 (4.3%)	0 (0.0%)	1 (4.8%)	51 (72.9%)	1 (4.3%)	4 (33.3%)	4 (25.0%)	0 (0.0%)	79 (24.5%)
Inherited	16 (44.4%)	7 (35.0%)	4 (40.0%)	4 (7.7%)	0 (0.0%)	5 (21.7%)	6 (37.5%)	6 (28.6%)	5 (7.1%)	10 (43.5%)	0 (0.0%)	3 (18.8%)	4 (40.0%)	70 (21.7%)
(*C*) *Cognition*														
Full-scale IQ														
N-Miss	8	2	1	4	1	2	1	0	9	2	5	4	2	41
Mean (s.d.)	80.964 (11.983)	73.389 (12.971)	91.667 (19.545)	76.542 (11.265)	81.667 (12.666)	79.381 (12.742)	78.200 (11.143)	86.286 (17.559)	74.902 (10.957)	86.476 (17.102)	60.143 (13.031)	80.417 (16.665)	82.000 (13.586)	78.811 (14.077)
Range	53.000–103.000	55.000–102.000	55.000–116.000	56.000–116.000	60.000–103.000	53.000–101.000	63.000–105.000	53.000–128.000	54.000–100.000	56.000–114.000	50.000–80.000	65.000–127.000	66.000–107.000	50.000–128.000
Performance IQ														
N-Miss	6	2	1	3	1	2	1	0	8	2	3	5	2	36
Mean (s.d.)	87.600 (13.992)	77.889 (12.778)	96.889 (18.537)	84.796 (11.923)	82.000 (11.878)	82.000 (10.950)	80.200 (9.221)	90.810 (16.482)	76.774 (10.659)	90.524 (20.707)	62.889 (12.036)	85.636 (17.408)	87.000 (14.031)	83.000 (14.695)
Range	57.000–124.000	56.000–106.000	62.000–115.000	63.000–119.000	64.000–104.000	58.000–102.000	69.000–103.000	58.000–126.000	57.000–106.000	57.000–127.000	53.000–84.000	69.000–132.000	72.000–111.000	53.000–132.000
Verbal IQ														
N-Miss	7	1	1	4	1	2	1	0	9	2	5	3	2	38
Mean (s.d.)	77.586 (11.425)	72.789 (12.809)	87.778 (17.448)	72.375 (12.006)	84.250 (12.664)	80.429 (17.066)	80.800 (15.599)	84.238 (17.490)	77.098 (12.416)	84.905 (13.707)	61.857 (11.725)	77.615 (15.441)	80.250 (13.833)	77.986 (14.354)
Range	55.000–103.000	55.000–101.000	55.000–113.000	55.000–110.000	63.000–101.000	55.000–107.000	60.000–120.000	55.000–123.000	55.000–100.000	62.000–112.000	55.000–80.000	58.000–117.000	64.000–101.000	55.000–123.000
(*D*) *Developmental behaviour checklist*														
Total behavioural problems score														
N-Miss	0	0	0	0	0	0	0	0	0	0	0	0	0	0
Mean (s.d.)	80.111 (29.871)	71.300 (26.160)	72.400 (41.762)	51.115 (24.463)	67.846 (26.655)	78.087 (27.624)	72.625 (31.256)	69.952 (21.388)	44.286 (26.107)	64.652 (32.109)	67.500 (21.000)	71.750 (19.485)	56.300 (28.414)	62.450 (29.622)
Range	16.000–130.000	27.000–125.000	17.000–139.000	2.000–102.000	34.000–108.000	17.000–121.000	13.000–157.000	19.000–108.000	4.000–115.000	10.000–121.000	31.000–99.000	32.000–105.000	9.000–94.000	2.000–157.000
Disruptive/antisocial subscale														
N-Miss	0	0	0	0	0	0	0	0	0	0	0	0	0	0
Mean (s.d.)	26.083 (13.622)	23.400 (10.995)	26.500 (15.953)	15.635 (8.682)	23.769 (11.300)	29.087 (12.007)	27.000 (11.989)	21.429 (9.442)	14.929 (10.220)	21.609 (12.752)	19.000 (9.420)	23.375 (9.598)	17.300 (9.934)	20.689 (11.901)
Range	7.000–49.000	8.000–43.000	3.000–47.000	1.000–38.000	12.000–44.000	4.000–48.000	5.000–53.000	6.000–39.000	1.000–42.000	2.000–45.000	6.000–33.000	9.000–47.000	2.000–30.000	1.000–53.000
Self-absorbed subscale														
N-Miss	0	0	0	0	0	0	0	0	0	0	0	0	0	0
Mean (s.d.)	25.139 (11.248)	23.200 (10.390)	23.300 (16.042)	15.712 (9.456)	20.769 (8.946)	24.087 (10.867)	22.500 (11.564)	22.571 (10.225)	12.843 (9.165)	21.435 (11.599)	25.500 (7.116)	25.125 (8.563)	16.900 (10.300)	19.708 (11.123)
Range	6.000–51.000	3.000–47.000	3.000–54.000	0.000–35.000	9.000–35.000	1.000–41.000	0.000–50.000	3.000–41.000	1.000–37.000	6.000–48.000	15.000–36.000	11.000–41.000	0.000–33.000	0.000–54.000
Communication disturbance subscale														
N-Miss	0	0	0	0	0	0	0	0	0	0	0	0	0	0
Mean (s.d.)	11.556 (4.398)	10.300 (4.473)	10.800 (5.653)	7.635 (3.881)	10.077 (6.184)	10.478 (4.450)	9.188 (6.014)	10.048 (3.775)	6.514 (4.089)	9.174 (6.322)	12.250 (4.224)	10.625 (4.440)	9.100 (5.626)	9.106 (4.907)
Range	1.000–21.000	2.000–18.000	1.000–19.000	0.000–16.000	1.000–18.000	1.000–18.000	0.000–24.000	2.000–16.000	0.000–17.000	0.000–21.000	3.000–17.000	1.000–18.000	0.000–19.000	0.000–24.000
Anxiety subscale														
N-Miss	0	0	0	0	0	0	0	0	0	0	0	0	0	0
Mean (s.d.)	9.611 (4.544)	7.450 (4.298)	6.100 (4.306)	6.558 (3.765)	6.077 (3.904)	8.826 (3.099)	7.562 (3.898)	7.619 (3.853)	5.886 (4.221)	6.870 (4.192)	5.333 (4.008)	7.125 (4.161)	8.300 (4.620)	7.115 (4.187)
Range	0.000–17.000	0.000–16.000	1.000–14.000	0.000–16.000	0.000–13.000	3.000–14.000	1.000–15.000	1.000–15.000	0.000–18.000	0.000–13.000	1.000–13.000	0.000–14.000	0.000–14.000	0.000–18.000
Social relating subscale														
N-Miss	0	0	0	0	0	0	0	0	0	0	0	0	0	0
Mean (s.d.)	7.722 (3.677)	6.950 (3.379)	5.700 (3.860)	5.577 (4.336)	7.154 (4.543)	5.609 (3.115)	6.375 (4.129)	8.286 (3.452)	4.114 (3.573)	5.565 (3.203)	5.417 (3.029)	5.500 (2.921)	4.700 (2.111)	5.832 (3.806)
Range	0.000–15.000	0.000–12.000	0.000–12.000	0.000–15.000	0.000–15.000	0.000–11.000	1.000–15.000	2.000–14.000	0.000–16.000	0.000–11.000	1.000–10.000	0.000–13.000	1.000–8.000	0.000–16.000
(*E*) *Health-Related variables*														
Participants taking psychiatric or epilepsy medication														
N-Miss	0	0	0	0	0	0	0	0	0	0	0	0	0	0
No	30 (83.3%)	19 (95.0%)	9 (90.0%)	45 (86.5%)	12 (92.3%)	17 (73.9%)	14 (87.5%)	19 (90.5%)	67 (95.7%)	20 (87.0%)	11 (91.7%)	13 (81.2%)	10 (100.0%)	286 (88.8%)
Yes	6 (16.7%)	1 (5.0%)	1 (10.0%)	7 (13.5%)	1 (7.7%)	6 (26.1%)	2 (12.5%)	2 (9.5%)	3 (4.3%)	3 (13.0%)	1 (8.3%)	3 (18.8%)	0 (0.0%)	36 (11.2%)
Congenital heart problems														
N-Miss	0	0	0	2	0	1	0	2	0	1	0	0	2	8
No	33 (91.7%)	18 (90.0%)	9 (90.0%)	45 (90.0%)	12 (92.3%)	19 (86.4%)	14 (87.5%)	15 (78.9%)	37 (52.9%)	17 (77.3%)	8 (66.7%)	13 (81.2%)	7 (87.5%)	247 (78.7%)
Yes	3 (8.3%)	2 (10.0%)	1 (10.0%)	5 (10.0%)	1 (7.7%)	3 (13.6%)	2 (12.5%)	4 (21.1%)	33 (47.1%)	5 (22.7%)	4 (33.3%)	3 (18.8%)	1 (12.5%)	67 (21.3%)
History of epileptic fits														
N-Miss	0	0	0	2	0	0	0	1	1	0	1	0	0	5
No	25 (69.4%)	18 (90.0%)	10 (100.0%)	36 (72.0%)	12 (92.3%)	23 (100.0%)	14 (87.5%)	15 (75.0%)	56 (81.2%)	21 (91.3%)	11 (100.0%)	10 (62.5%)	9 (90.0%)	260 (82.0%)
Yes	11 (30.6%)	2 (10.0%)	0 (0.0%)	14 (28.0%)	1 (7.7%)	0 (0.0%)	2 (12.5%)	5 (25.0%)	13 (18.8%)	2 (8.7%)	0 (0.0%)	6 (37.5%)	1 (10.0%)	57 (18.0%)
Born prematurely (<37 Weeks)														
N-Miss	2	2	1	2	0	4	1	3	4	0	0	0	2	21
Early	16 (47.1%)	9 (50.0%)	5 (55.6%)	22 (44.0%)	6 (46.2%)	9 (47.4%)	12 (80.0%)	7 (38.9%)	34 (51.5%)	11 (47.8%)	5 (41.7%)	9 (56.2%)	4 (50.0%)	149 (49.5%)
Not Early	18 (52.9%)	9 (50.0%)	4 (44.4%)	28 (56.0%)	7 (53.8%)	10 (52.6%)	3 (20.0%)	11 (61.1%)	32 (48.5%)	12 (52.2%)	7 (58.3%)	7 (43.8%)	4 (50.0%)	152 (50.5%)
Child part of multiple birth														
N-Miss	1	1	0	2	0	2	2	4	2	0	0	0	0	14
No	34 (97.1%)	19 (100.0%)	10 (100.0%)	49 (98.0%)	13 (100.0%)	21 (100.0%)	14 (100.0%)	16 (94.1%)	66 (97.1%)	23 (100.0%)	12 (100.0%)	15 (93.8%)	10 (100.0%)	302 (98.1%)
Yes	1 (2.9%)	0 (0.0%)	0 (0.0%)	1 (2.0%)	0 (0.0%)	0 (0.0%)	0 (0.0%)	1 (5.9%)	2 (2.9%)	0 (0.0%)	0 (0.0%)	1 (6.2%)	0 (0.0%)	6 (1.9%)
Fertility treatment used during pregnancy														
N-Miss	2	2	0	5	0	2	2	4	2	0	0	0	0	19
No	30 (88.2%)	17 (94.4%)	10 (100.0%)	42 (89.4%)	13 (100.0%)	21 (100.0%)	13 (92.9%)	17 (100.0%)	65 (95.6%)	22 (95.7%)	11 (91.7%)	15 (93.8%)	10 (100.0%)	286 (94.4%)
Yes	4 (11.8%)	1 (5.6%)	0 (0.0%)	5 (10.6%)	0 (0.0%)	0 (0.0%)	1 (7.1%)	0 (0.0%)	3 (4.4%)	1 (4.3%)	1 (8.3%)	1 (6.2%)	0 (0.0%)	17 (5.6%)
Prolonged labour														
N-Miss	3	3	1	3	1	3	2	4	3	0	0	0	0	23
No	27 (81.8%)	15 (88.2%)	9 (100.0%)	44 (89.8%)	12 (100.0%)	16 (80.0%)	12 (85.7%)	14 (82.4%)	59 (88.1%)	20 (87.0%)	11 (91.7%)	14 (87.5%)	8 (80.0%)	261 (87.3%)
Yes	6 (18.2%)	2 (11.8%)	0 (0.0%)	5 (10.2%)	0 (0.0%)	4 (20.0%)	2 (14.3%)	3 (17.6%)	8 (11.9%)	3 (13.0%)	1 (8.3%)	2 (12.5%)	2 (20.0%)	38 (12.7%)
Birth by caesarean section														
N-Miss	1	1	1	2	0	2	3	4	2	1	0	0	0	17
No	23 (65.7%)	16 (84.2%)	8 (88.9%)	31 (62.0%)	8 (61.5%)	15 (71.4%)	10 (76.9%)	8 (47.1%)	49 (72.1%)	12 (54.5%)	9 (75.0%)	12 (75.0%)	9 (90.0%)	210 (68.9%)
Yes	12 (34.3%)	3 (15.8%)	1 (11.1%)	19 (38.0%)	5 (38.5%)	6 (28.6%)	3 (23.1%)	9 (52.9%)	19 (27.9%)	10 (45.5%)	3 (25.0%)	4 (25.0%)	1 (10.0%)	95 (31.1%)
Child spent time in a special care baby unit														
N-Miss	1	2	1	4	0	3	2	4	4	1	1	1	0	24
No	31 (88.6%)	15 (83.3%)	7 (77.8%)	43 (89.6%)	12 (92.3%)	17 (85.0%)	11 (78.6%)	14 (82.4%)	40 (60.6%)	16 (72.7%)	8 (72.7%)	13 (86.7%)	9 (90.0%)	236 (79.2%)
Yes	4 (11.4%)	3 (16.7%)	2 (22.2%)	5 (10.4%)	1 (7.7%)	3 (15.0%)	3 (21.4%)	3 (17.6%)	26 (39.4%)	6 (27.3%)	3 (27.3%)	2 (13.3%)	1 (10.0%)	62 (20.8%)
Child spent time in an incubator														
N-Miss	2	2	1	3	0	2	3	4	3	0	0	1	0	21
No	33 (97.1%)	12 (66.7%)	7 (77.8%)	43 (87.8%)	12 (92.3%)	18 (85.7%)	10 (76.9%)	16 (94.1%)	43 (64.2%)	17 (73.9%)	7 (58.3%)	13 (86.7%)	9 (90.0%)	240 (79.7%)
Yes	1 (2.9%)	6 (33.3%)	2 (22.2%)	6 (12.2%)	1 (7.7%)	3 (14.3%)	3 (23.1%)	1 (5.9%)	24 (35.8%)	6 (26.1%)	5 (41.7%)	2 (13.3%)	1 (10.0%)	61 (20.3%)

### A high percentage of young people with ND-CNVs screen positive for clinically significant difficulties on the DBC and family income plays a role

In total, 223 of 322 (69.3%) individuals with an ND-CNV screened positive for clinically significant difficulties. Logistic regression showed this was predicted by family income (OR = 0.71, CI = 0.55–0.91, *p* = 0.008, *p*_adj_ = 0.025) with children from families with higher incomes being less likely to experience clinically significant difficulties. Age, gender and maternal education level were not associated with the presence of clinically significant difficulties.

### No gender differences

A chi-squared test revealed that there was no difference between the number of males (156/214, 72.9%) or females (67/108, 62.0%) that screened positive for clinically significant difficulties (χ^2^ = 3.48, *p* = 0.062, *p*_adj_ = 0.131). However, we found a nominally significant difference in self-absorbed score, with males scoring higher than females (*t* = 2.10, *p* = 0.037, *p*_adj_ = 0.083). Mean total problems score, or mean scores on any other subscale did not differ between males and females.

### Health and pregnancy-related variables are not associated with rates of clinically significant difficulties in young people with ND-CNVs

We examined the influence of the following variables on the presence of clinically significant difficulties: the presence of congenital heart defects; a history of epileptic fits; being part of a multiple births; whether the mother had fertility treatment during the pregnancy; or prolonged labour (>36 h); the baby having spent time in a special care baby unit (SCBU); or in an incubator; or having been born prematurely (before 37 weeks for a single birth, or before 34 weeks for twins). The model also included age, gender, maternal education level and approximate family income as covariates. None of these health or pregnancy-related variables was associated with screening positive or negative for clinically significant difficulties. This remained the case after excluding individuals taking psychiatric or epilepsy medication.

### ID-associated emotions and behaviours that are more or less common in young people with ND-CNVs

In order to investigate which emotions and behaviours are more or less common in individuals with ND-CNVs, we plotted the deviation from the mean for each item and each ND-CNV genotype in a heatmap (Fig. 1). For each subscale, the mean total severity scores for each question were calculated and then ranked from lowest to highest. The items with the highest mean severity scores were ‘easily led by others’ on the disruptive/antisocial scale, ‘doesn't mix well with own age group’ on the communication disturbance scale, ‘poor sense of danger’ on the self-absorbed scale, ‘tends to be a loner’ on the social relating scale and ‘fears particular things or situations’ on the anxiety scale. The lowest mean severity scores were seen for ‘lights fires’ on the disruptive/antisocial scale, ‘overly interested in mechanical things’ on the communication disturbance scale, ‘bites others’ on the self-absorbed scale, ‘Sleeps too much’ on the social relating scale, and ‘loss of appetite’ on the anxiety scale. Full results are shown in Fig. 1. Questions that remained significant after correction for multiple comparisons are indicated by red points in each upper panel.

### ND-CNV genotypes differ in risk for clinically significant difficulties on the DBC

**Fig. 2.**
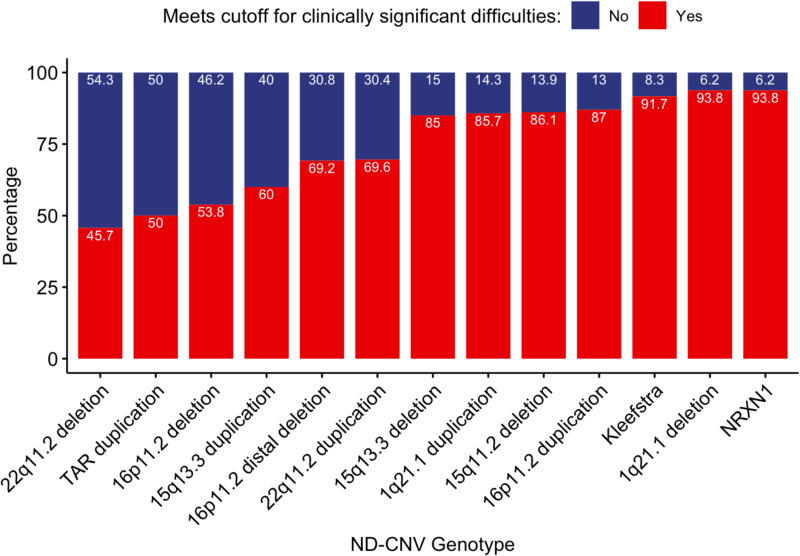
Percentages of individuals screening positive and negative for clinically significant difficulties (DBC total problems score >45).

To investigate if ND-CNV genotypes differ in risk for clinically significant difficulties (based on total DBC cut-off), we performed a likelihood ratio test comparing models where the presence of difficulties was predicted by family income with a model where ND-CNV genotype was also included. The addition of ND-CNV genotype improved prediction of the presence of clinically significant difficulties (df = 12, χ^2^ = 39.99, *p* = <0.001, *p*_adj_<0.001). Figure 2. shows the percentage of individuals displaying clinically significant difficulties by ND-CNV genotype. Rates of clinically significant difficulties differed across genotypes (χ^2^ = 51.20, df = 12, *p* < 0.001, *p*_adj_ = <0.001). Rates of screening positive ranged from 45.7% (32/70) in 22q11.2 deletion to 93.8% (15/16) in individuals with 1q21.1 deletion. ND-CNV genotype remained a significant predictor after excluding individuals taking psychiatric or epilepsy medication (df = 12, χ^2^ = 36.58, *p* < 0.001, *p*_adj_ = 0.001).

ND-CNV genotype was associated with TBPS and all subscales (Table 2). Table 2 also presents the variance in the TBPS and subdomain scores that is explained by ND-CNV genotype (final column, *η*_p_^2^). This was found to range from 9.3% for anxiety to 15.9% of the self-absorbed score, along with genotype explaining 16.4% of the variance in TBPS. There remained a significant effect of ND-CNV genotype for all scores when individuals taking psychiatric or epilepsy medication were excluded.

**Table 2.** Variance explained by ND-CNV genotype on (A) DBC total problems score, and (B–F) subscale scores, with family income as a covariate

**(A) DBC total problems**	***F***	***p***	***p*_adj_**	***η*_p_^2^**
ND-CNV	4.45	<0.001	<0.001	0.164
Family Income	1.09	0.352	0.454	0.012
**(B) Disruptive/antisocial**				
ND-CNV	3.85	<0.001	<0.001	0.146
Family Income	1.05	0.371	0.472	0.011
**(C) Communication disturbance**				
ND-CNV	3.08	<0.001	<0.001	0.120
Family Income	0.64	0.591	0.643	0.007
**(D) Self-absorbed**				
ND-CNV	4.28	<0.001	<0.001	0.159
Family Income	1.17	0.322	0.430	0.013
**(E) Social relating**				
ND-CNV	3.09	<0.001	<0.001	0.120
Family Income	1.01	0.388	0.484	0.011
**(F) Anxiety**				
ND-CNV	2.32	0.008	0.024	0.093
Family Income	0.67	0.571	0.628	0.007

We also used ANCOVAs to investigate if having an inherited (*n* = 79) or *de novo* (*n* = 70) ND-CNV (173 individuals had unknown inheritance) or if the type of genomic change (deletion or duplication) affected TBPS or subscale scores, with family income as a covariate. These tests revealed that having an inherited ND-CNV was associated with greater TBPS (*F* = 7.46, df = 1, *p* = 0.007, *p*_adj_ = 0.022, *η*_p_^2^ = 0.053), disruptive/antisocial score (*F* = 4.64, *p* = 0.033, *p*_adj_ = 0.075, *η*_p_^2^ = 0.034), self-absorbed score (*F* = 4.69, df = 1, *p* = 0.032, *p*_adj_ = 0.074, *η*_p_^2^ = 0.034), communication disturbance score (*F* = 9.20, df = 1, *p* = 0.003, *p*_adj_ = 0.011, *η*_p_^2^ = 0.065) and anxiety score (*F* = 5.11, df = 1, *p* = 0.025, *p*_adj_ = 0.059, *η*_p_^2^ = 0.037) but not social relating score. However, inheritance was also strongly associated with ND-CNV genotype (χ^2^ = 90.85, df = 12, Cramer's *V* = 0.781, *p* = <0.001, *p*_adj_<0.001), and including both ND-CNV genotype and inheritance status in the model resulted in inheritance no longer being significantly associated with greater problems. Type of genomic change was not associated with TBPS or scores on any of the subscales.

**Fig. 3.**
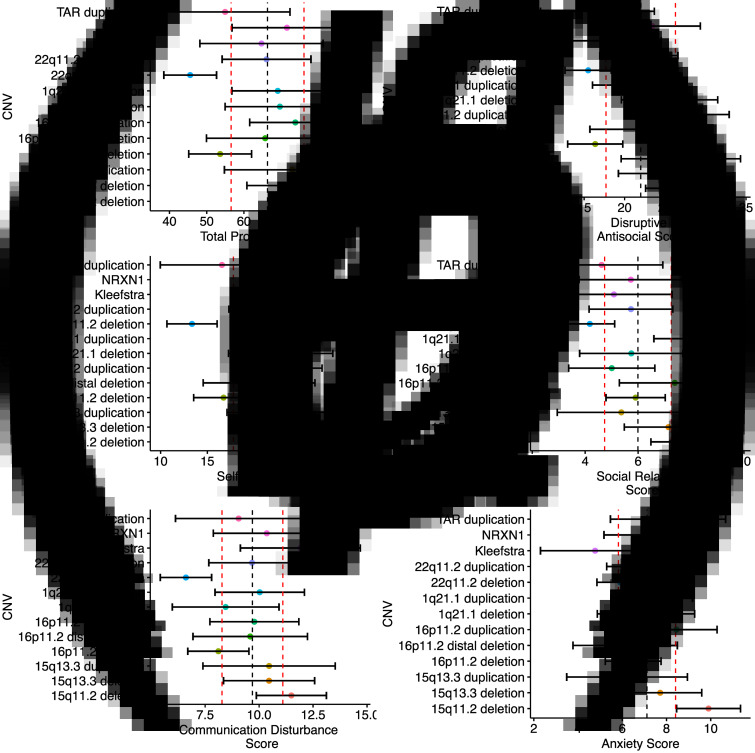
Marginal mean scores for each ND-CNV genotype on (*a*) DBC total problems score and (*b–f*) the five subscales. The overall group mean and 1 standard deviation above and below are indicated by the dashed lines. Error bars indicate 95% confidence intervals of the estimated marginal mean. Marginal means are estimated from ANCOVAs including family income as a covariate.

The patterns of severity across DBC subdomains between the ND-CNV genotypes can be seen in Fig. 3, which plots marginal mean scores on the TBPS and each of the subscales. These marginal mean scores represent the predicted score after adjusting for family income. Using a Friedman's Chi-squared test, we found that there was a significant difference in the severity of scores depending on ND-CNV genotype (Friedman χ^2^ = 40.09, df = 12, *p* < 0.001, *p*_adj_<0.001). Kendall's coefficient of concordance revealed that there was also high concordance between scores on the subscales (*F* = 6.28, *p* < 0.001, *p*_adj_<0.001) with a large effect size (*W* = 0.56).

## Discussion

This is the first study, as far as we are aware, that compares phenotypes between a range of different ND-CNV genotypes using a measure of emotion and behaviour problems designed for young people with ID. We demonstrate that emotion and behaviour difficulties are highly prevalent across the ND-CNV genotypes we studied, with 69.3% of young people with ND-CNVs screening positive for clinically significant difficulties. ND-CNV genotype was associated with both screening positive for clinically significant difficulties, as well as total behaviour problems score (TBPS), suggesting that the ND-CNV genotypes display different levels and patterns of difficulties. We found that ND-CNV genotype explained approximately 16.4% of the variance in TBPS and between 7 and 16% of the variance in subscale scores. Type of genomic change (deletion or duplication), did not affect DBC scores. Our models suggested that children with inherited ND-CNVs did display greater overall difficulties, and greater levels of disruptive/antisocial problems, self-absorbed behaviours, communication disturbance, and anxiety problems than children with *de novo* variants. However, inheritance status and ND-CNV genotype were significantly linked, reflecting that rates of inheritance differed between ND-CNV genotypes. We were therefore not able to evaluate the impact of inheritance status independently.

Previous studies have described high rates and complex presentations of psychiatric and neurodevelopmental difficulties in individuals with ND-CNVs (Chawner et al., 2019; Niarchou et al., 2014; Schneider et al., 2014; Steinman et al., 2016) using standard psychiatric assessments. Our findings, using a measure designed for use in ID that focuses on emotion and behaviour disturbance, offers further evidence for elevated rates of psychopathology in individuals with ND-CNVs.

We found no evidence of gender differences in the rate of clinically significant difficulties. This agrees with previous research using the DBC in populations with intellectual disability (Einfeld & Tonge, 1996; Koskentausta & Almqvist, 2004). However, we did find that screening positive for emotion and behaviour problems was associated with family income, suggesting that a higher income might be protective against problems with emotion and behaviour in young people with ND-CNVs. It will be important to further explore the mechanisms underlying this protective effect.

Within the ND-CNV sample we found that scores on aggressive and problematic conduct behaviour such as ‘lights fires’, ‘bites others’, ‘steals’ were consistently lowered compared to the average, while questions that might indicate difficulties with socialising such as ‘doesn't mix with own age group’, ‘poor sense of danger’ or ‘tends to be a loner’; along with questions related to mood and attention, such as ‘cries easily’, ‘becomes overexcited’, ‘has temper tantrums’, ‘impatient’ or ‘impulsive’ were consistently found to be more severe than the average. This might suggest that broadly speaking, children with ND-CNVs show fewer problems with aggressive behaviour or conduct problems, but may need extra support around socialising and mood problems alongside attention and hyperactivity symptoms.

In the present study, we also find differences in both screening positive for difficulties and in severity scores depending on ND-CNV genotype. Rates of positive screening for clinically significant difficulties ranged from 45.7% in young people with 22q11.2 deletion to 93.8% in young people with 1q21.1 deletion. The implication of this is that some ND-CNV genotypes are more likely to display problems with emotion and behaviour than others. This is also supported by our findings of high concordance of severity scores for the ND-CNV genotypes between the DBC scales, which suggests that those ND-CNVs that score low on one scale also score low on the other scales, and vice versa. However, ND-CNV genotype explained only a low proportion of variance in TBPS and subscale severity scores. This suggests that there are other factors that contribute to psychopathology in these individuals. These could include background genetic risk (potentially measured by a polygenic risk score) (Cleynen et al., 2019) and environmental risk factors, such as bullying or lack of family support. Indeed, we find that approximate family income is predictive of rates of clinically significant difficulties, in agreement with evidence that lower socioeconomic status is associated with greater rates of childhood multimorbidity (Cornish, Boyd, Van Staa, Salisbury, & Macleod, 2013; Johnson, Cornish, Boyd, & Macleod, 2019).

This is the largest study of its kind to investigate patterns of emotion and behaviour problems across different ND-CNV genotypes. However, there are differences in the size of samples of individuals with different ND-CNV genotypes. We were also unable to account for familial liability for behavioural problems as the DBC is not suited for assessments in unaffected familial members, who are unlikely to display ID. While we found potential evidence that individuals with inherited ND-CNVs displayed greater difficulties, this was confounded by the strong association between inheritance and ND-CNV genotype. We can therefore not draw any conclusions about the effect of inheritance on behavioural problems.

Ascertainment bias may affect our results, as the developmental delay is a major reason for referral for genetic testing in the UK. This might be highlighted by our findings of individuals with 15q11.2 deletion having some of the most severe scores on the DBC, despite continued debate over the clinical significance of this ND-CNV genotype (Butler, 2017), along with the relatively lower problems displayed by individuals with 22q11.2 deletion, who are more likely to be referred to genetics clinics for medical issues. It may be that the full range of outcomes (including no or mild phenotypic effects) are not captured in this study. However, even if we are predominantly including those who are also more likely to have a higher load of background genetic and environmental risk factors, it is still important to better understand the difficulties faced by this group of patients, as they make up a significant proportion of those presenting for clinical genetic testing and care following a CNV diagnosis.

Clinicians such as genetic counsellors or paediatricians often lack complete information on the likely outcomes of patients with an ND-CNV in their care and are therefore unable to provide accurate counselling or advice to patients or their families. Improving our knowledge and understanding of emotion and behaviour risk is, therefore, key to improving counselling provision and access to appropriate services. This is particularly relevant as numbers of patients presenting for genetic screening will increase as the technology used to detect chromosomal aberrations continues to improve.

### Clinical implications

Immediate clinical implications of these findings should be increased vigilance for emotion and behaviour problems in individuals with ND-CNVs. If ID is suspected, then psychiatric assessments that are designed to be used in individuals with ID should be applied. Particular difficulties in individuals with ND-CNVs may be poor social skills, attention difficulties and mood problems. However, it would be best for every child to complete a DBC so that interventions can be targeted at an individual level. In addition, patients who display both ID and emotion or behaviour problems, in the absence of any physical injury that could cause ID, are potential candidates for genetic screening for contributory genetic variants. Individuals from lower-income backgrounds may be at greater risk for clinically significant difficulties with emotions and behaviour.

## Conclusions

Emotion and behaviour difficulties are common in individuals with ND-CNVs. These problems may show specific patterns depending on ND-CNV genotype, but individuals with ND-CNVs show particular problems with social relating, attention, impulsivity and fearing particular things or situations. It is important that future research involving young people with ND-CNVs includes measures adapted for ID such as the DBC.

## Supplementary material

For supplementary material accompanying this paper visit https://doi.org/10.1017/S0033291720002330. click here to view supplementary material
