# Supplementary material for: Assessment of emotions and behaviour by the Developmental Behaviour Checklist in young people with neurodevelopmental CNVs
Source: Psychol Med. 2020 Jul 9;52(3):574–86. doi: 10.1017/S0033291720002330 (PMC7794095; doi:10.1017/S0033291720002330)
Supplement: Supplementary file 1 [file S0033291720002330sup.zip › S0033291720002330sup001.docx]

Supplementary Table 2. Variance explained by ND-CNV genotype on A) DBC total problems score, and B-F) subscale scores, with family income as a covariate and medication users excluded from the sample.

| **A) DBC total problems** | **F** | **p** | **η_p_^2^** |
| --- | --- | --- | --- |
| ND-CNV | 3.46 | 0.000 | 0.148 |
| Family Income | 1.27 | 0.261 | 0.005 |
| **B) Disruptive/antisocial** | **F** | **p** | **η_p_^2^** |
| ND-CNV | 2.88 | 0.001 | 0.126 |
| Family Income | 1.49 | 0.223 | 0.006 |
| **C) Communication disturbance** | **F** | **p** | **η_p_^2^** |
| ND-CNV | 2.61 | 0.003 | 0.115 |
| Family Income | 0.10 | 0.751 | 0.000 |
| **D) Self-absorbed** | **F** | **p** | **η_p_^2^** |
| ND-CNV | 3.37 | 0.000 | 0.144 |
| Family Income | 1.40 | 0.237 | 0.006 |
| **E) Social relating** | **F** | **p** | **η_p_^2^** |
| ND-CNV | 2.85 | 0.001 | 0.125 |
| Family Income | 0.66 | 0.416 | 0.003 |
| **F) Anxiety** | **F** | **p** | **η_p_^2^** |
| ND-CNV | 1.99 | 0.025 | 0.091 |
| Family Income | 0.05 | 0.824 | 0.000 |
