# Supplementary material for: Assessment of emotions and behaviour by the Developmental Behaviour Checklist in young people with neurodevelopmental CNVs
Source: Psychol Med. 2020 Jul 9;52(3):574–86. doi: 10.1017/S0033291720002330 (PMC7794095; doi:10.1017/S0033291720002330)
Supplement: Supplementary file 1 [file S0033291720002330sup.zip › S0033291720002330sup002.docx]

**IMAGINE-ID Consortium Membership**

^1^ MRC Centre for Neuropsychiatric Genetics and Genomics, Division of Psychological Medicine and Clinical Neurosciences, Cardiff University School of Medicine, Cardiff, UK.

^2^ School of Clinical Medicine, University of Cambridge, Cambridge Biomedical Campus, Cambridge, UK

^3^ NIHR BRC Great Ormond Street Institute of Child Health, University College London, London, UK

^4^ Unique – The Rare Chromosome Disorder Support Group, London, UK.

Sophie Andrews^1^, Philippa Birch^1^, Samantha Bowen^1^, Karen Bradley^1^, Dr. Samuel Chawner^1^, Dr. Andrew Cuthbert^1^, Prof. Jeremy Hall^1^, Sarah Law^1^, Nicola Lewis^1^, Dr. Sinead Morrison^1^, Hayley Moss^1^, Prof. Sir. Michael Owen^1^, Sinead Ray^1^, Matthew Sopp^1^, Molly Tong^1^, Prof. Marianne van den Bree^1^, Dr Kate Baker^2^, Eleanor Dewhurst^2^, Amy Lafont^2^, Prof. Lucy Raymond^2^, Terry Shirley^2^, Hayley Tilley^2^, Husne Timur^2^, Catherine Titterton^2^, Neil Walker^2^, Sarah Wallwork^2^, Francesca Wicks^2^, Dr Zheng Ye^2^, Marie Erwood^2^, Spiros Denaxas^3^, Hayley Denyer^3^, Nasrtullah Fatih^3^, Manoj Juj^3^, Ellie Kerry^3^, Anna Lucock^3^, Frida Printzlau^3^, Prof. David Skuse^3^, Dr Ramya Srinivasan^3^, Alice Watkins^3^, Jeanne Wolstencroft^3^, Beverly Searle^4^

**IMAGINE-ID Clinical Collaborators**

| **Title (if applicable)** | **First Name** | **Surname** | **Shorthand Institution** | **Genetics service** | **Hospital Trust** |
| --- | --- | --- | --- | --- | --- |
| Dr | John | Dean | Aberdeen | Aberdeen Royal Infirmary Genetics Service | NHS Grampian |
| Dr | Lisa | Robertson | Aberdeen | Aberdeen Royal Infirmary Genetics Service | NHS Grampian |
| Dr | Denise | Williams | Birmingham | West Midlands Regional Genetics Service | Birmingham Women's NHS Foundation Trust |
| Dr | Alan | Donaldson | Bristol | Bristol Clinical Genetics Service | University Hospitals Bristol NHS Foundation Trust |
| Professor | Lucy | Raymond | Cambridge | East Anglian Medical Genetics Service | Cambridge University Hospitals NHS Foundation trust |
| Dr | Annie | Procter | Cardiff | All Wales Regional Genetics Service | Cardiff and Vale University LHB |
| Dr | Jonathan | Berg | Dundee | Ninewells Hospital Dundee Genetics Service | NHS Tayside |
|  | Yanick | Crow | Edinburgh | Western General Hospital Edinburgh Genetics Service | NHS Lothian |
| Professor | Anne | Lampe | Edinburgh | Western General Hospital Edinburgh Genetics Service | NHS Lothian |
| Dr | Julia | Rankin | Exeter | Peninsula Genetics Service | Royal Devon and Exeter NHS Foundation Trust |
| Dr | Shelagh | Joss | Glasgow | Glasgow Genetics Centre | NHS Greater Glasgow & Clyde |
| Professor | Lyn | Chitty | GOSH | London North East Thames Regional Genetics Service - Clinical Genetics | Great Ormond Street Hospital for Children NHS Foundation Trust |
| Professor | Frances | Flinter | Guy's | London Guy's Hospital Genetic Centre | Guy's and St. Thomas' NHS Foundation Trust |
| Dr | Muriel | Holder | Guy's | London Guy's Hospital Genetic Centre | Guy's and St. Thomas' NHS Foundation Trust |
| Dr | Alison | Kraus | Leeds | Yorkshire Regional Genetics Service - Clinical Genetics | Leeds Teaching Hospitals NHS Trust |
| Dr | Julian | Barwell | Leicester | Leicestershire Genetics Centre | University Hospitals of Leicester NHS Trust |
| Dr | Pradeep | Vasudevan | Leicester | Leicestershire Genetics Centre | University Hospitals of Leicester NHS Trust |
| Dr | Astrid | Weber | Liverpool | Cheshire & Merseyside Regional Genetic Service | Liverpool Women's NHS Foundation Trust |
| Dr | William | Newman | Manchester | Manchester Centre for Genomic Medicine | Central Manchester University Hospitals NHS Foundation trust |
| Dr | Miranda | Splitt | Newcastle | Northern Genetics Service | The Newcastle upon Tyne Hospitals NHS Foundation Trust |
| Dr | Virginia | Clowes | North West Thames | London North West Thames Regional Genetics Service | North West London Hospitals NHS Trust |
| Dr | Fleur | van Dijk | North West Thames | London North West Thames Regional Genetics Service | North West London Hospitals NHS Trust |
| Dr | Rachel | Harrison | Nottingham | Nottingham Regional Genetics Service | Nottingham University Hospitals NHS Trust |
| Dr | Usha | Kini | Oxford | Oxford Genetics Service | Oxford University Hospitals NHS Trust |
| Dr | Oliver | Quarrell | Sheffield | Sheffield Genetic Services | Sheffield Children's NHS Foundation Trust |
| Dr | Diana | Baralle | Southampton | Wessex Clinical Genetics Service | University Hospital Southampton NHS Foundation Trust |
| Dr | Sahar | Mansour | St George's | London South West Thames Regional Genetics Service | St George’s Healthcare NHS Foundation Trust |
