# Supplementary material for: Assessment of emotions and behaviour by the Developmental Behaviour Checklist in young people with neurodevelopmental CNVs
Source: Psychol Med. 2020 Jul 9;52(3):574–86. doi: 10.1017/S0033291720002330 (PMC7794095; doi:10.1017/S0033291720002330)
Supplement: Supplementary file 1 [file S0033291720002330sup.zip › S0033291720002330sup003.docx]

Supplementary Table 1. Psychiatric and epilepsy medications being used in sample.

| Medication | Number of individuals |
| --- | --- |
| Amphetamine/dextroamphetamine | 1 |
| Aripiprazole | 1 |
| Atomoxetine | 4 |
| Carbamazepine | 2 |
| Clobazam | 1 |
| Ethosuximide | 1 |
| Fluoxetine | 2 |
| Lamotrigine | 2 |
| Levetiracetam | 2 |
| Lithium | 1 |
| Methylphenidate | 7 |
| Nitrazepam | 1 |
| Risperidone | 2 |
| Sertraline | 3 |
| Sodium Valproate | 10 |
| Topiramate | 1 |
| Unspecified ADHD Medication | 1 |
| Unspecified Epilepsy Medication | 1 |
